# Levels, patterns and determinants of using reversible contraceptives for limiting family planning in India: evidence from National Family Health Survey, 2015–16

**DOI:** 10.1186/s12905-022-01706-0

**Published:** 2022-04-19

**Authors:** Margubur Rahaman, Risha Singh, Pradip Chouhan, Avijit Roy, Sumela Ajmer, Md Juel Rana

**Affiliations:** 1 Department of Migration and Urban Studies, International Institute for Population Sciences (IIPS), Mumbai, 400088 India; 2 Centre for the Study of Regional Development (CSRD), Jawaharlal Nehru University, New Delhi, 110067 India; 3 Department of Geography, University of Gour Banga (UGB), Malda, 732103 India; 4 Department of Geography, University of Gour Banga (UGB), Malda, 732103 India; 5 Department of Geography, Tilka Manjhi Bhagalpur University, Bhagalpur, 812001 India; 6 College of Health Science, Korea University, Seoul, 028401 South Korea

**Keywords:** Limiting demand, Demand satisfied, Reversible contraception, Family planning, India

## Abstract

**Background:**

Demand for family planning is predominantly for birth limiting rather than birth spacing in India. Despite several family planning programmes in India, the use of reversible contraception for limiting family planning has been stagnant and largely depends on female sterilization. Though many researchers have examined patterns and determinants of using modern contraception for total family planning, studies on patterns and determinants of contraceptive use for birth limiting are limited in India. This paper examines the patterns of contraceptive use for liming demand and its determinants in India.

**Methods:**

The National Family Health Survey-4, 2015–16 data was used. Bivariate chi-square significant test and multivariate binary logistic regression model used to accomplish the study objectives.

**Results:**

Majority of women (86.5%) satisfied limiting demand (SLD) in India; the SLD was found significantly low among the women’s age 15–19 years (53.1%) and parity 0 (42%). The satisfied limiting demand by modern reversible contraception (mrSLD) was found significantly high in age group 15–19 years (49.1%), Muslims (30.6%) and North-east region (45.4%). The satisfied limiting demand by traditional contraception (tSLD) was almost three times higher in North-east region (26.1%) than national average of India (8.7%). The women’s years of schooling, wealth status, religion and presence of son child found to be significant determinants of mrSLD. The likelihood of tSLD was found significantly high among the women who had no son child (AOR = 1.41; 95% CI:1.34, 1.48), Muslim (AOR = 1.78; 95% CI:1.70, 1.87). A considerable regional variability in levels of SLD, mrSLD and tSLD was found in India.

**Conclusion:**

Public investment in family planning is required to promote and provide subsidized modern reversible contraception (MRC) services, especially to women from North-east region, Muslim, Scheduled tribe, poor household and who had no son child. Improving the quality and availability of MRC services in public health centre will be helpful to increase SLD among the above mentioned women. Besides, the promotion of MRC will be supportive to overcome the issues of sterilization regrets in India.

**Supplementary Information:**

The online version contains supplementary material available at 10.1186/s12905-022-01706-0.

## Background

The practice of ideal family planning is positively linked with the socio-economic development of society and individuals. The demand for family planning means when individuals want to stop their childbearing permanently (limiting demand) or delay for atleast two or more years (spacing demand) [1]. The demand significantly depends on individuals’ fertility behaviour and socio-economic backgrounds. Since 1951, India has implemented several programmes to increase family planning acceptance and couples choice-based contraceptive method use [2–4]. However, the long history of family planning in India suggested that the practice of family planning is primarily tilted towards birth limiting rather than spacing [5]. In addition, the demand for limiting childbearing has mainly been met by using permanent contraceptives [4]. The total demand and using contraceptives (met need) for limiting childbearing are remarkably varied across Indian states and union territories [6].

India has achieved noteworthy progress in reducing the fertility rate over the recent period. However, some states in central and north India are still lagged behind from desire goal [7]. Recent National Family Health Survey (NFHS-4) data (2015–16) shows that about 83.3% of women had limiting demand out of total demand for family planning (66%) in India [7]. Furthermore, the demand for family planning for limiting childbearing was five times higher than spacing in India in 2015–16 [7]. Accordingly, the success of family planning programme has been highly depending on the using modern contraception method to satisfy limiting demand in India. If the unmet need (not using any contraceptives demand) or use of traditional contraception methods to satisfy limiting demand will increase, then it can be alarming issue for high populated country India.

A number of studies have examined the trends, patterns, and determinants of unmet need for total family planning in India and elsewhere [4, 8–11]. However, no previous studies have examined the unmet need and use of contractive methods for limiting demand in India based on NFHS-4 (2015–16) data. In this context, the present study aims to examine the patterns and determinants of demand satisfied for limiting childbearing among currently married women, limiting demand satisfied with modern reversible contraception, and limiting demand satisfied with traditional contraception in India.

## Methods

### Data

**Fig. 1:**
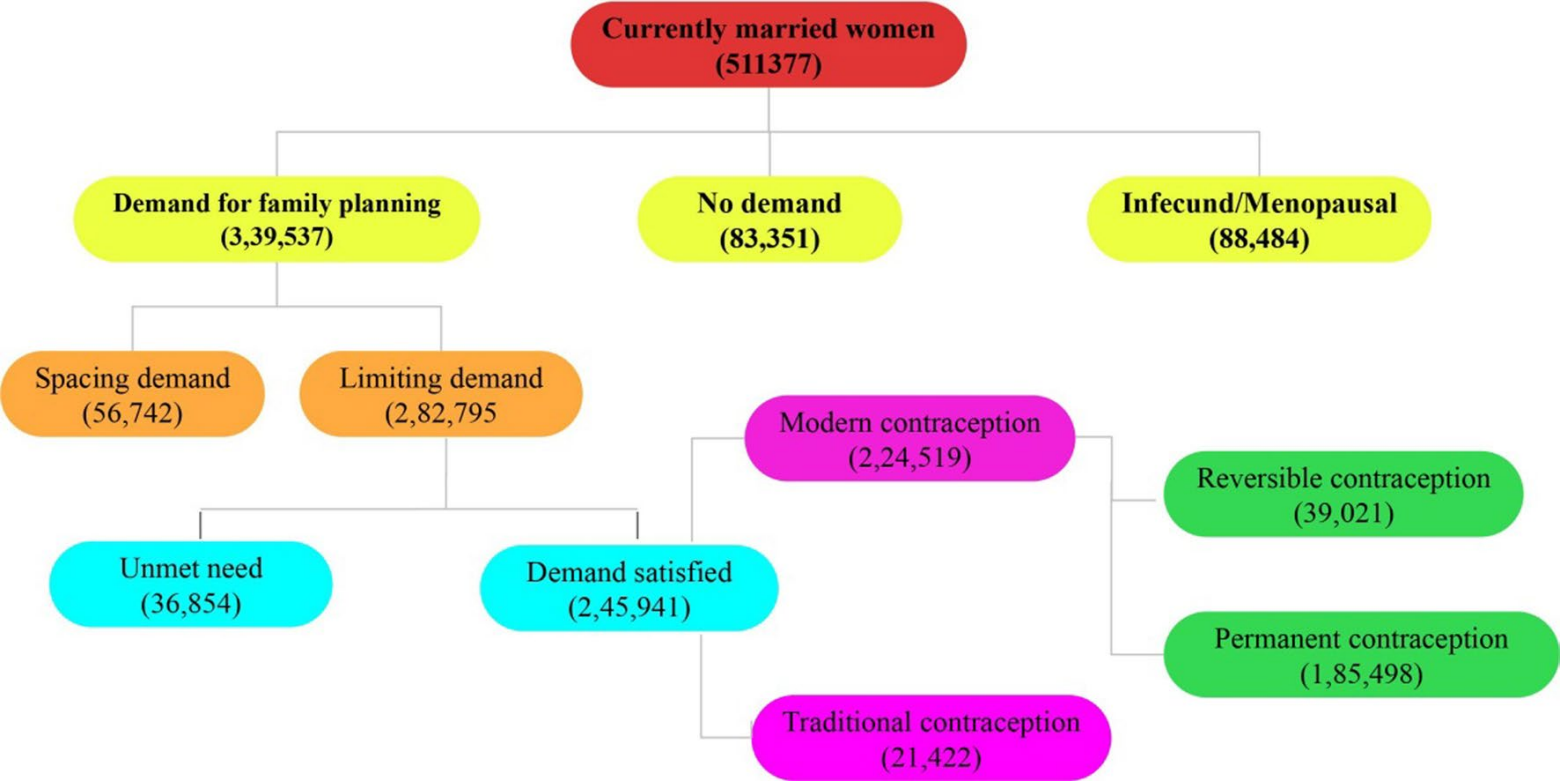
Chart presentation of currently married women who had demand for limiting family planning with type of using contraception in India, 2015–16

The study has used unit level data from the fourth round of the National Family Health Survey (NFHS 4), carried out during 2015–16. This survey adopts two-stage stratified systematic random sampling. In the first stage, the primary sample unit has been selected and the household has been selected in the second stage. This survey has collected information for the representation of the districts of India. The main objectives of the survey are to provide reliable estimates on fertility, maternal and child mortality, family planning, reproductive and child health, nutritional status of children, utilization of maternal and child health care services, and women’s autonomy. The survey adopted the multistage sampling design to collect the samples [7]. The NFHS collected data using different interview schedules–household schedule and eligible women, men and biomarker schedule. The NFHS 4 survey had interviewed total 699,686 women aged 15–49 years in India. The paper limits the sample to those who were currently married women during the survey period. After excluding unmarried, divorced, separated, and widow, the total sample used for analysis is 511,377 currently married women. Among the total currently married women, total demand for family planning includes 339,537 women and demand for limiting includes 282,795 women. The details of the using sample distribution have displayed in Fig. 1. Among the women who have demand for limiting methods, the demand satisfied for limiting includes 245,941 women (185,498 by modern permanent method, 39,021 by modern reversible method and 21,422 by traditional methods).

### Outcome variables

The NFHS 4 collected information on demand and using contraception method for family planning. The outcome variables have been prepared based on the following questions. “Do you want to limit your pregnancy or childbearing for permanently?” If yes, then it is called limiting demand. If women have limiting demand, the next question for them: “Are you using any contraceptives to limit your pregnancy for permanently?” If answer is yes, then it called ‘demand satisfied for limiting’ otherwise ‘unmet need for limiting’.

The selected outcome variables are (a) demand satisfied for limiting childbearing (SLD) (b) limiting demand satisfied by modern reversible contraception methods (mrSLD) (c) limiting demand satisfied by traditional contraception methods (tSLD). Here, SLD denotes when women were using any contraception methods to satisfy their limiting demand. The mrSLD means when women were using modern reversible contraception methods (pill, IUD, injectable, male condom, female condom, standard days method (SDM), diaphragm, foam/jelly, lactational amenorrhoea method (LAM), and other modern methods) to satisfy their limiting demand. And, tSLD refers when women were using traditional contraception methods (rhythm, withdrawal, and other traditional methods) to satisfy limiting demand.

### Explanatory variables

The explanatory variable used in the analyses includes a range of socioeconomic and demographic variables, which have been significantly associated with unmet and met need for family planning in India and elsewhere (8–11). These independent variables are- respondent's age (15–19, 20–24, 25–29, 30–34, 35 and above years), parity (0, 1, 2, 3, 4+), have at least one son (had at least one son [Yes], had no son [No]), women's years of schooling (No schooling, 1–5 years, 6–10 years, 11 and above years), wealth status (poorest, poorer, middle, richer and richest), religion (Hindu, Muslim, Christians and other) and social group (General, other backward class [OBC], Scheduled Caste [SC], Scheduled Tribe [ST] and Don’t know), mass media exposure (had not listening family planning programme through mass-media [No], had listening family planning programme through mass-media [Yes]), place of residence (urban, rural) and geographical region (North, Central, East, North-East, West, and South). The division of geographical regions was taken from the NFHS-4 report [9].

### Statistical analysis

Bivariate and multivariate analysis which includes cross-tabulation and binary logistic regression was used to accomplish the study objectives. Bivariate analysis with Chi-square significance test was applied to examine the patterns of the limiting demand, satisfied limiting, and using contraceptive method by those who satisfied limiting demand. Three separate binary logistic regression analyses were performed to determine the socio-economic correlates of SLD, mrSLD and, tSLD. These variables are binary in nature like SLD (Unmet need ‘0’, Demand satisfied ‘1’), mrSLD (No ‘0’; Yes ‘1’), and tSLD (No ‘0’; Yes ‘1’). The study used revised definitions of unmet need for limiting [12]. The results of binary logistic regression analysis were presented in the form of adjusted odds ratios (AOR) with a 95% confidence interval (CI). All analyses were carried out using statistical STATA (version 14.1 SE).

## Results

### Background characteristics of sample

Table 1 presents background characteristics of the selected currently married women. Almost 7% of women belong from early reproductive age group (15–19 years). Almost 20% of women responded their parity was four and above. A significant number of women were poorest (18.2%) and with no schooling (33.2%). Majority of women were Hindus (81.4), followed by Muslims (13.2). Almost 43% of women belong from other backward class (OBCs). Majority of women (62.7%) were listening mass media. Two third women (66.6%) were belongs from rural area.

**Table 1:** Background characteristics of currently married women in India, 2015–16

Background characteristics	n	Percent
Age group		
15–19	18,492	3.6
20–24	80,275	15.7
25–29	102,715	20.1
30–34	90,898	17.8
35+	218,997	42.8
Parity		
0	50,878	10.0
1	93,286	18.2
2	167,107	32.7
3	100,701	19.7
4+	99,406	19.4
Have at least one son		
Yes	377,210	73.8
No	134,168	26.2
Years of schooling		
No education	169,603	33.2
1–5 years	72,319	14.1
6–10 years	171,980	33.6
11 and above years	97,476	19.1
Wealth status		
Poorest	92,872	18.2
Poorer	100,897	19.7
Middle	104,683	20.5
Richer	107,485	21.0
Richest	105,440	20.6
Religion		
Hindu	416,467	81.4
Muslim	67,308	13.2
Christian	11,371	2.2
Others	16,231	3.2
Social group		
General	114,654	22.4
OBC	223,224	43.7
SC	103,603	20.3
ST	46,698	9.1
Don’t know	23,199	4.5
Mass media exposure		
No	190,800	37.3
Yes	320,577	62.7
Place of residence		
Urban	170,870	33.4
Rural	340,507	66.6
Region		
North	68,625	13.4
Central	115,369	22.6
East	118,153	23.1
North-east	17,335	3.4
West	73,716	14.4
South	118,178	23.1
N	511,377	100

###  Levels of limiting demand

Table 2 presents the levels of limiting demand among current married women (15–49 years) by selected socio-demographic background in India during 2015–16. The limiting demand was 86.5% in India. In addition, it was noticeably varied with socio-background. It was increased with women’s age and parity. Majority of women started to limit their childbearing at age 35 and above (73.3%) and parity 3 (74.4%). The limiting demand was almost three times higher among the women who had at least one son (67.6%) than who had no son child (20.6%). The gap in limiting demand was significantly visible between no schooling (59.6%) and years of schooling 11 or above (44.4%), and between poorest (48.5%) and richest (58.2%). Among the religious and social group group, the limiting demand was slightly low among Muslims (47.1%) and STs (51.1%). The regional variability in limiting demand had ranges from 43.2% in North-east to 61.2% in North.

**Table 2:** Distribution of demand for family planning by background characteristics of the currently married women aged 15–49 years in India, 2015–16 (n = 511,377)

Background characteristics	Demand for family planning (%)				Chi^2^ significance test
	Spacing	Limiting	No demand	Infecund and menopausal	
Age group					
15–19	32.2	4.8	59.7	3.3	*P* < 0.001
20–24	29.4	21.8	44.3	4.5	
25–29	17.7	48.9	23.8	9.6	
30–34	7.2	67.5	9.6	15.7	
35–39	1.1	73.3	1.6	27.5	
Parity					
0	17.6	2.1	59.8	20.6	*P* < 0.001
1	34.3	20.4	29.8	15.5	
2	6.3	71.5	8.3	13.9	
3	3.2	74.4	6.0	16.4	
4+	2.1	68.7	5.2	24.0	
Have at least one son					
Yes	7.0	67.6	8.0	17.4	*P* < 0.001
No	22.6	20.6	39.6	17.1	
Years of schooling					
No schooling	5.4	59.6	11.8	23.2	*P* < 0.001
1–5 years	8.3	61.2	13.9	16.6	
6–10 years	12.8	54.8	17.8	14.6	
11 and above years	20.1	44.4	23.2	12.4	
Wealth status					
Poorest	10.3	48.5	20.7	20.5	*P* < 0.001
Poorer	11.1	53.9	17.5	17.5	
Middle	10.3	57.0	16.0	16.7	
Richer	11.1	58.0	14.6	16.4	
Richest	12.6	58.2	13.4	15.8	
Religion					
Hindu	10.5	56.4	15.9	17.3	*P* < 0.001
Muslim	14.6	47.1	19.7	18.6	
Christians	11.6	52.6	15.9	20.0	
Others	13.0	63.4	13.0	10.7	
Social group					
General	12.0	58.6	13.8	15.7	*P* < 0.001
OBC	10.4	54.6	16.8	18.3	
SC	10.7	56.3	16.9	16.2	
ST	11.3	51.1	20.0	17.6	
Don’t know	15.1	50.4	14.4	20.2	
Mass media exposure					
No	9.8	53.1	17.0	20.1	*P* < 0.001
Yes	11.9	56.6	15.9	15.6	
Place of resident					
Urban	11.4	57.9	13.9	16.8	*P* < 0.001
Rural	10.9	54.0	17.5	17.6	
Region					
North	12.1	61.2	15.3	11.5	*P* < 0.001
Central	11.5	52.6	18.9	17.1	
East	13.8	49.1	16.9	20.3	
Northeast	21.4	43.2	15.0	20.5	
West	10.1	60.6	14.8	14.5	
South	6.6	59.2	14.9	19.2	
India	11.1	55.3	16.3	17.3	
n	56,742	282,795	83,351	88,484	

### Levels of satisfied limiting demand (SLD)

Table 3 presents the socio-economic differentials in SLD in India during 2015–16. The percentage of SLD was significantly low among the women age group 15–19 years (53.1%) and parity 0 (42%). It was slightly higher among the women who had at least one son child (87.6%) compared to those who had no son child (81%). Among the religious group, the SLD was found comparatively low in Muslims (80.2%) than others (91%). The SLD had somewhat lower among STs (84.8%) compared to national average (86.5%). The rural–urban gap in SLD was negligible. The SLD was found significantly lower in North-east (80%), Central (82.2%), and East (83%) compared to South region (93.1%).

**Table 3:** Levels of demand satisfied for limiting family planning (SLD) among currently married women (15–49 years) those who had demand for limiting by background characteristics, India 2015–16

Background characteristics	n	Demand satisfied (%)	Unmet need (%)	Chi^2^ significance test
Age group				
15–19	892	53.1	46.9	*P* < 0.001
20–24	17,493	70.0	30.0	
25–29	50,243	79.0	21.0	
30–34	61,378	86.1	13.9	
35 and above	152,790	92.1	7.9	
Parity				
0	1,052	42.0	58.0	*P* < 0.001
1	19,004	76.9	23.1	
2	119,497	88.5	11.5	
3	74,915	89.4	10.6	
4+	68,327	85.0	15.0	
Have at least one son				
Yes	255,109	87.6	12.4	*P* < 0.001
No	27,686	81.0	19.0	
Years of schooling				
No education	101,076	87.3	12.7	*P* < 0.001
1–5 years	44,238	89.0	11.0	
6–10 years	94,248	87.3	12.7	
11 and above years	43,234	83.3	16.7	
Wealth status				
Poorest	45,044	79.3	20.7	*P* < 0.001
Poorer	54,404	86.6	13.4	
Middle	59,673	89.2	10.8	
Richer	62,283	88.9	11.1	
Richest	61,392	88.8	11.2	
Religion				
Hindu	234,818	87.7	12.3	*P* < 0.001
Muslim	31,712	80.2	19.8	
Christians	5979	88.7	11.3	
Others	10,287	91.0	9.0	
Social group				
General	67,167	87.2	12.8	*P* < 0.001
OBC	121,794	86.5	13.5	
SC	58,285	88.1	11.9	
ST	23,867	84.8	15.2	
Don’t know	11,683	86.7	13.3	
Mass media exposure				
No	101,264	84.3	15.7	*P* < 0.001
Yes	181,531	88.5	11.5	
Place of resident				
Urban	98,889	87.8	12.2	*P* < 0.001
Rural	183,906	86.5	13.5	
Region				
North	42,011	88.9	11.1	*P* < 0.001
Central	60,644	82.2	17.8	
East	57,995	83.0	17.0	
Northeast	7487	80.1	19.9	
West	44,672	88.4	11.6	
South	69,986	93.1	6.9	
India	282,795	86.5	13.5	

### Using contraceptive method-mix for SLD

Table 4 shows the levels of using contraceptive methods among the women who satisfied limiting demand (SLD) with background characteristics in India, 2015–16. Result shows that the majority of women were using modern permanent (75.4%) compared to modern reversible (15.9%) and traditional contraception (7.8%) method in India. The use of modern permanent contraception was increased with increasing women’s age and parity. Similarly, it was decreased with increasing women’s years of schooling and wealth status. Religious differentials in using modern permanent contraception method was significantly existed, ranges from 55.4% in Muslims to 86.8% in Christians. Similarly, the gap between General and ST was also significant, ranges from 65.9 to 83.7%, respectively. Nevertheless, rural–urban gap was 7.2%, in rural (78%) and urban (70.8%). A wide regional gap in using modern permanent contraception found between North-east (28.5%) and South (97.2%).

**Table 4:** Patterns of contraceptive method-mix among the currently married women (15–49 years) who satisfied limiting demand, India 2015–16 (n = 245,940)

Background characteristics	n	Modern contraception (%)		Tradition contraception (%)	Chi^2^ significance test
		Permanent	Reversible		
Age group					
15–19	474	34.3	49.1	16.6	*P* < 0.001
20–24	12,245	60.2	29.8	10.0	
25–29	39,672	67.0	23.8	9.2	
30–34	52,867	70.4	20.4	9.2	
35 and above	140,683	81.2	10.6	8.2	
Parity					
0	442	53.9	28.5	17.7	*P* < 0.001
1	14,605	39.1	40.0	20.9	
2	105,798	76.1	16.8	7.1	
3	66,988	81.4	12.0	6.6	
4+	58,108	76.7	12.4	10.9	
Have at least one son					
Yes	223,523	76.2	15.3	8.5	*P* < 0.001
No	22,418	67.6	21.5	10.9	
Years of schooling					
No education	88,265	82.7	9.2	8.2	*P* < 0.001
1–5 years	39,356	78.9	13.3	7.8	
6–10 years	82,296	73.1	18.2	8.8	
11 and above years	36,023	59.3	29.9	10.9	
Wealth status					
Poorest	35,697	76.3	12.4	11.3	*P* < 0.001
Poorer	47,115	76.3	14.3	9.4	
Middle	53,211	79.6	12.6	7.7	
Richer	55,377	77.6	15.0	7.4	
Richest	54,540	67.7	23.6	8.7	
Religion					
Hindu	205,862	78.0	13.8	8.2	*P* < 0.001
Muslim	25,417	55.4	30.6	14.0	
Christians	5302	86.8	8.6	4.6	
Others	9360	67.5	24.8	7.7	
Social group					
General	58,584	65.9	23.6	10.5	*P* < 0.001
Other backward class	105,396	79.5	12.6	7.9	
Scheduled caste	51,352	78.3	13.4	8.3	
Scheduled tribe	20,699	83.7	10.1	6.2	
Don’t know	9910	56.0	30.1	13.9	
Mass media exposure					
No	85,321	77.4	12.8	9.7	*P* < 0.001
Yes	160,620	74.4	17.5	8.2	
Place of resident					
Urban	86,860	70.8	20.5	8.7	*P* < 0.001
Rural	159,081	78.0	13.4	8.7	
Region					
North	37,346	65.9	25.2	9.0	*P* < 0.001
Central	49,871	63.5	19.3	17.3	
East	48,137	64.4	22.8	12.8	
North East	5997	28.5	45.4	26.1	
West	39,469	84.1	12.6	3.3	
South	65,121	97.2	2.1	0.7	
India	245,940	75.4	15.9	8.7	

The use of modern reversible contraception to satisfy limiting demand was noticeably high among the women of age group 15–19 years (49.1%), and who had single parity (40%) years of schooling 11 years or above (29.9%), richest (23.6%), Muslims (30.6%), and general caste (23.6%). The use of modern reversible contraception was varied from 45.1% in North-east to 2.1 in South. The use of traditional contraception method was more than two times higher than national average (8.2%) found among the women of age group 15–19 years (16.6%), of single parity (20.9%), Muslims (14%), in north-east region (26.1) and central region (17.3%).

### Results from logistic regression models

#### Satisfied limiting demand (SLD)

The logistic regression model after adjusting the effects of predictors revealed that women’s age, parity, having son child, years of schooling, wealth status, religion, social group, mass media exposure, place of residence, and the region continued to be significant determinants of SLD (Table 5). The likelihood of the SLD was significantly increased with increasing women’s age, parity and wealth status. Similar result also found in unadjusted model (Additional file 1). The SLD was found 65% (AOR:1.65; 95% CI:1.42–1.92) more likely in age group 25–29 years compared to the age group 15–19 years. The likelihood of SLD was almost five-fold higher in women whose parity was two (AOR: 5.37; 95% CI:4.65–6.21) compared to those having zero parity. The adjusted odds of SLD found 0.68 less likely among the women who had not at least one son (AOR: 0.68; 95% CI:0.65–0.70) as compared to women who had son. The women with 1–5 years of schooling were 12% (AOR = 1.12, 95% CI:1.07–1.16) more likely satisfied limiting demand (SLD) compared to women with no schooling. However, the odds of SLD found slightly low (AOR = 0.93, 95% CI:0.89–0.95) among the women with 11 or more years of schooling compared to no schooling, similar result also found in unadjusted regression model (Additional file 1). The richest women were 78% more likely to have their limiting demand satisfied compared to the poorest women. The likelihood of SLD was 42% (AOR = 0.58, 95% CI:0.56–0.60) less likely in Muslims compared to Hindu. Women who belonged to OBCs were 15% (AOR = 0.85, 95% CI:0.83–0.88) less likely to SLD as compared to the General category. Further, a positive association between mass media usage and met need for limiting childbearing (SLD) was observed. The women who had mass media exposure related to family planning were 30% more likely to be using contraceptives for limiting childbearing compared to women who had no exposure. Women from rural areas (AOR: 1.11; 95% CI:1.08–1.15) were more likely to SLD than women in urban areas. The likelihood of SLD was found almost two times higher (OR: 1.90; 95% CI:1.81–1.99) in South compared to the North region. The odds of SLD was found lower in North-east (AOR = 0.67; 95% CI:0.63–0.72), Central (AOR = 0.71; 95% CI:0.68–0.74) and East (AOR = 0.83; 95% CI:0.80–0.87) compared to the North region.

**Table 5:** Adjusted odds ratio (AOR) of the SLD, mrSLD and tSLD among the currently married women (15–49 years) with limiting demand by background characteristics in India, 2015–16

Variables	SLD	mrSLD	tSLD
	AOR [95% CI]	AOR [95% CI]	AOR [95%CI]
Age group			
15–19 (ref.)	1.00	1.00	1.00
20–24	1.06 [0.91,1.24]	0.65*** [0.52,0.81]	1.19 [0.90,1.56]
25–29	1.65*** [1.42,1.92]	0.47*** [0.38,0.59]	1.25 [0.95,1.64]
30–34	2.92*** [2.5,3.40]	0.36*** [0.29,0.45]	1.28 [0.98,1.67]
35+	5.45*** [4.68,6.34]	0.17*** [0.14,0.22]	1.28 [0.98,1.68]
Parity			
0 (ref.)	1.00	1.00	1.00
1	2.88*** [2.49,3.33]	2.20*** [1.69,2.86]	1.40* [1.06,1.85]
2	5.37*** [4.65,6.21]	0.96 [0.74,1.25]	0.52*** [0.39,0.68]
3	5.16*** [4.46,5.97]	0.74* [0.57,0.96]	0.39*** [0.30,0.51]
4+	3.26*** [2.82,3.78]	0.90 [0.7,1.18]	0.51*** [0.39,0.67]
Have at least one son			
Yes (ref.)	1.00	1.00	1.00
No	0.68*** [0.65,0.7]	1.37*** [1.31,1.43]	1.41*** [1.34,1.48]
Years of schooling			
No education (ref.)	1.00	1.00	1.00
1–5 years	1.12*** [1.07,1.16]	1.28*** [1.23,1.33]	1.06 [1.01,1.11]
6–10 years	0.84*** [0.81,0.87]	1.83*** [1.76,1.90]	1.43*** [1.37,1.49]
11 and above years	0.53*** [0.51,0.55]	3.36*** [3.2,3.51]	1.68*** [1.59,1.78]
Wealth status			
Poorest (ref.)	1.00	1.00	1.00
Poorer	1.53*** [1.48,1.59]	1.16*** [1.11,1.21]	0.94 [0.90,0.99]
Middle	1.75*** [1.68,1.82]	1.11*** [1.06,1.17]	0.98 [0.93,1.03]
Richer	1.69*** [1.61,1.76]	1.24*** [1.18,1.31]	0.95 [0.90,1.01]
Richest	1.78*** [1.69,1.88]	1.52*** [1.43,1.61]	0.86*** [0.81,0.92]
Religion			
Hindu (ref.)	1.00	1.00	1.00
Muslim	0.58*** [0.56,0.6]	3.15*** [3.03,3.27]	1.78*** [1.70,1.87]
Christian	0.70*** [0.64,0.76]	1.32*** [1.18,1.48]	1.34 [0.69,1.55]
Others	1.23*** [1.15,1.33]	1.32*** [1.24,1.39]	0.97 [0.89,1.05]
Social group			
General (ref.)	1.00	1.00	1.00
OBC	0.85*** [0.83,0.88]	0.83*** [0.81,0.86]	0.96 [0.92,1]
SC	1.02 [0.98,1.06]	0.91*** [0.88,0.95]	1.01 [0.97,1.06]
ST	1.09*** [1.03,1.14]	0.69*** [0.65,0.73]	0.63 [0.59,1.68]
Don’t know	1.14*** [1.07,1.21]	1.01 [0.95,1.07]	0.97 [0.9,1.04]
Mass media exposure			
No (ref.)	1.00	1.00	1.00
Yes	1.27*** [1.24,1.31]	1.04*** [1.01,1.08]	0.80*** [0.78,0.83]
Place of residence			
Urban (ref.)	1.00	1.00	1.00
Rural	1.11*** [1.08,1.15]	0.71*** [0.69,0.73]	0.88*** [0.85,0.91]
Region			
North (ref.)	1.00	1.00	1.00
Central	0.71*** [0.68,0.74]	0.93*** [0.89,0.96]	2.27*** [2.17,2.38]
east	0.83*** [0.8,0.87]	0.94*** [0.91,0.98]	1.27*** [1.21,1.33]
North-east	0.67*** [0.63,0.72]	2.58*** [2.41,2.75]	2.94*** [2.73,3.17]
West	1.03 [0.98,1.08]	0.35*** [0.33,0.36]	0.30*** [0.28,0.33]
South	1.90*** [1.81,1.99]	0.05*** [0.05,0.05]	0.06*** [0.05,0.07]
n	282,795	245,941	245,941
Log likelihood	− 98,327.045	− 83,591.163	− 62,154.098
LR chi^2^	22,228.47	47,986.56	21,179.94
Prob > chi^2^	< 0.001	< 0.001	< 0.001
Pseudo R^2^	0.1016	0.0223	0.1456

***< 0.001; **< 0.01; *< 0.05; 95% confidence interval (CI) have been presented in the parentheses

#### Modern reversible contraceptive use for SLD (mrSLD)

Women’s age, parity, years of schooling, wealth status and, religion and social group were found significant socio-economic correlates of mrSLD in India (Table 5). The odds of mrSLD were decreased with increasing women’s age and parity. The likelihood of mrSLD was found 1.4 times (AOR: 1.37; 95% CI:1.31–1.43) more likely among the women who had not son as compared to the women who had at least one son. Both unadjusted and adjusted odds of mrSLD confirmed a positive association between mrSLD and women’s years of schooling and wealth status. The woman with 11 or more years of schooling shows higher odds of mrSLD (AOR: 3.36; 95% CI:3.20–3.51) compared to the reference group i.e. no schooling (Additional file 1). Similarly, women in the richest wealth group were 1.5 times (AOR: 1.52; 95% CI:1.43–1.61) more likely to be mrSLD than the poorest. The likelihood of mrSLD was 3.15 (AOR = 3.15; 95% CI:3.03–3.27), 1.32 (AOR = 1.32; 95% CI:1.18–1.48), and 1.32 (AOR = 1.32; 95% CI:1.24–1.39) times more likely found in Muslims, Christians, and others respectively compared to reference group i.e. Hindu. The mrSLD was significantly determined by status of social group. The likelihood of mrSLD was found significantly lowest (AOR = 0.69; CI:0.65–0.73) among STs compared to general caste. Women living in rural areas (AOR = 0.71; 95% CI:0.69–0.73) were less likely to be using modern reversible contraceptives to limiting childbearing (mrSLD) compared to women of urban areas. The likelihood of mrSLD was found 2.58 (AOR = 2.58; 95% CI:2.41–2.75) times higher in North-East region as compared to the North region. However, it was considerably low in West (AOR = 0.35; 95% CI:0.33–0.36) and South (AOR = 0.05; 95% CI:0.03–0.07) as compared to the North region.

#### Traditional contraceptive use for SLD (tSLD)

The women’s years of schooling, wealth status, religion, caste, mass media exposure, place of residence, and region was significantly associated with tSLD (Table 5). There was no significant association between tSLD and women’s age in adjusted model; however, the association was found significant in unadjusted model (Additional file 1). The odds of tSLD was found significantly high among the women with single parity (AOR = 1.40; 95% CI:1.06–1.85) compared to zero parity. The women who completed 11 or more years of schooling were more likely satisfied limiting demand (AOR = 1.68; 95% CI:1.59–1.78) by using traditional contraceptives (tSLD) as compared to women with no schooling. However, the likelihood of tSLD was found 22% (AOR = 0.86; 95% CI:0.81–0.92) less likely among richest women than the poorest ones. The odds of tSLD was found remarkably high among Muslim women (AOR = 1.78; 95% CI:1.70–1.87) compared to Hindu. The women with mass media exposure were less likely use tSLD (AOR = 0.80; 95% CI:0.78–0.83) compared to those who do not have exposure to mass media. Rural women have a less likelihood (AOR = 0.88; 95% CI:0.85–0.91) of using tSLD than the urban counterpart. A huge regional variability in likelihood of tSLD was found in India. Further, the likelihood of tSLD was found 2.94 (AOR = 2.94; 95% CI:2.73–3.17), 2.27 (AOR = 2.27; 95% CI:2.17–2.38) and 1.27 (AOR = 1.27; 95% CI:1.21–1.33) higher in North-east, Central and East region, respectively as compared to women in North region.

## Discussion

This study presents an assessment of the patterns and determinants of using contraception for limiting family planning in India. Our study found that family planning demand predominantly for birth limiting rather than birth spacing among across the socio-economic groups, except early reproductive and low parity women in India; several previous studies also suggested similar results. Modern permanent contraception continued to be the dominant method used to satisfy the limiting demand. It’s happened due to promotion and availability of subsidised and incentives by family welfare department for male and female sterilization. On the other hand, the limited use of modern reversible contraception for limiting found primarily due to quality issue, side-effect, out of pocket expenditure and lack of knowledge about alternative modern contraception [13, 14]. However, almost one-tenth of women were using traditional contraception to meet limiting demand in India. Even after two and half decades of the recommendations from the International Conference on Population and Development (ICPD) in 1994, India has been introduced several couple-centric contraception or “basket of contraceptives programme” to ensure couple choice based family planning programme. Therefore, the persistent low use of reversible contraception raised questions on the quality of basket of contraceptive programme.

The study found that limiting demand was significantly low among the women in early reproductive age group (15–19 years), with low parity (0–1 child), Muslim and North-East region. The demand was found low among the women in early reproductive age group and with single parity may be due to desire of more children [15]. Some Muslim women believed in religious prohibition of limiting family planning, which somewhat increased the demand for spacing family planning among them [16]. The high demand for spacing rather than limiting family planning in North-east region suggested there is need a study to explore it.

Demand satisfied for limiting (SLD) was found noticeably low found among the women in age group 15–19 and 20–24 years. This finding is similar with previous study [8]. The presence of high unmet need will be increase the probability of unwanted births and spontaneous abortion among them [8]. Likewise, the women who had not at least one son were less likely satisfied limiting demand. Many previous studies also suggested that the unmet need for family planning significantly higher among them [17]. It indicates son child preference negatively associated with family planning in India.

Women’s years of schooling and wealth status positively associated with mrSLD found in our study. With the improvement in the educational level, it is expected that women become more aware of family planning and knowledge about alternative contraceptives [18–20]. The wealth condition reflects the affordability and purchasing power of contraceptive methods of family planning [21, 22]. The likelihood of mrSLD was decreased with increasing women’s age and parity. It’s possibly because with progression in age and parity, women achieve desired number of children and they do not want to take risk of method failure of reversible contraception. Muslim women more likely used modern reversible contraception may be due to religious barriers. Previous many studies suggested that rigid religious belief is the main reason for not-using permanent contraceptive methods among Muslims [16, 23]. The poor-rich, illiterate-educated, General-ST and, rural–urban gap in mrSLD suggested that there is huge difference in knowledge, affordability and accessibility of modern reversible contraception among them. Lower accessibility of modern reversible method and less information about contraceptive methods and low follow-ups are the main significant barriers to extensive use of reversible methods in rural areas [17, 24]. High level of socio-economic deprivation, remoteness and lack of knowledge about modern reversible contraception are main reason of low mrSLD among STs. The women who had not at least son child primarily inclined towards modern reversible contraception for limiting childbearing; it is because of modern reversible contraception gives them opportunity to exit from limiting demand and became pregnant in future desired to achieve son child. The likelihood of mrSLD was noticeably higher among women in North-east suggested that there is need to ensure the public supply of modern reversible contraception. Other hand, the limited use of modern reversible methods in South and West region suggested that limited choice of contraception availability. The extreme use of permanent method for limiting child in South and West region has been increasing sterilization regrets found in previous studies [25]. Mass media played important role to increase use of modern reversible contraception found in our study, the finding similar with previous studies [4, 10, 11].

The use of traditional contraception to satisfy limiting demand (tSLD) decreased with increasing women’s parity and wealth status. The wealthier and multiparous women mainly used modern reversible contraception also found in previous studies [4, 9, 26]. The higher educated women (11 or more years of schooling) were more likely to tSLD compared to no schooling. This finding is analogous with previous studies [9]. It may occur due to quality and supply issues of modern reversible contraception or maybe they know properly how to control pregnancy by traditional way. The likelihood of tSLD was found high among the women who had no children and Muslim. Some previous studies also suggested that the prevalence of traditional contraception significantly high among them [4, 9, 27–29]. Previous studies suggested that due to erroneous religious hearsay, a sizable portion of backward Muslim communities adopted traditional spacing contraception instead of modren contraception [9, 28]. Except South and West region, the likelihood of tSLD was found significantly high compared to North. The use of traditional contraception extremely high found in Central, East and North-east region also found by previous study [4, 9, 10]. The tSLD was particularly in these regions positively linked with socio-economic backwardness (higher poverty, low women's education and autonomy), lack of knowledge about contraception method-mix, and poor quality and covearge of family planning services [28].

Major findings of the study and policy recommendation-

- Firstly, the modern permanent contraception continued to be the dominant method used to satisfy the limiting demand suggested that there is need to promote modern reversible contraception, especially long acting reversible contraception (IUD, injectable) in India. It will be minimize the risk of sterilization regrets in India.
- Secondly, the women who had no son child, belongs from Muslim and OBCs, resides in Central, East and North-east region less likely satisfied limiting demand. Government should focus on these women to reduce the unmet need for limiting family planning in India.
- Thirdly, the poor-rich, uneducated-educated, General-ST and rural–urban gap in mrSLD suggested that there is need to increase the availability, affordability and quality of modern reversible contraception in India.
- Fourthly, the use of modern reversible contraception for limiting demand significantly high found among the women in early reproductive age, with single parity, had no son child, belong to Muslim and North-east region. The government should ensure the supply of modern reversible contraception for above targeted groups.

### Strengths and limitations

The primary strength of this study is a complete analysis of limiting family planning in India by using nationally representative data. This study will be helpful to understand the patterns of demand for limiting family planning, contraception-method mix and associated factors. In addition, the patterns and determinants of modern reversible and traditional contraception using for limiting demand also examined. This study has competent to capture the target groups who were lagged behind in terms of limiting family planning. These will be helpful for policy maker and researcher to extend the research on family planning and policy review. The limitation of the study is this study presents only national level scenario, there is need to further analysis on district level analysis to capture the micro-level issues of family planning in India.

## Conclusion

The satisfied limiting demand was noticeably low among the women in early reproductive age group, had no son child, Muslim; which suggested that government should focus on the above groups. The limiting demand mainly satisfied by modern permanent contraception in India raised voice on women’s contraception choice and reproductive health rights. The promotion of reversible contraception for limiting childbearing is needed to be communicated at the policy-level. There is need to supply the modern contraception among the women who were using traditional contraception for limiting family planning. The study highlights that ministry of family and health welfare (MoFHW) needs to pay attention to promote the client choice-based contraceptives availability, accessibility, and affordability to reduce the unmet need for family planning and sterilization regrets in India. The promotion of couple choice-based contraceptive methods would help to achieve the sustainable development goals of ensuring the reproductive health and rights of all girls and women.

## Supplementary Information

**Additional file 1: Appendix Table 1.** The unadjusted odds ratio (uOR) of the demand satisfied (SLD), use of modern reversible methods (mrSLD) and traditional methods (tSLD) by background characteristics in India, 2015-16.

## Data Availability

The data can be downloaded through online upon a granted request from Demographic Health Survey (https://dhsprogram.com/data/available-datasets.cfm).

## Abbreviation

SLD: Satisfied limiting demand
mrSLD: limiting demand satisfied by modern reversible contraception
tSLD: limiting demand satisfied by traditional contraception
NFHS: National Family Health Survey
AOR: Adjusted odds ratio
CI: Confidence interval
