# Supplementary material for: Levels, patterns and determinants of using reversible contraceptives for limiting family planning in India: evidence from National Family Health Survey, 2015–16
Source: BMC Womens Health. 2022 Apr 19;22:124. doi: 10.1186/s12905-022-01706-0 (PMC9020013; doi:10.1186/s12905-022-01706-0)
Supplement: Supplementary file 1 — Additional file 1: Appendix Table 1. The unadjusted odds ratio (uOR) of the demand satisfied (SLD), use of modern reversible methods (mrSLD) and traditional methods (tSLD) by background characteristics in India, 2015-16. [file 12905_2022_1706_MOESM1_ESM.docx]

Appendix

Table 1: The unadjusted odds ratio (uOR) of the demand satisfied (SLD), use of modern reversible methods (mrSLD) and traditional methods (tSLD) by background characteristics in India, 2015-16

| Variables | Demand satisfied |  | Modern reversible |  | Traditional |
| --- | --- | --- | --- | --- | --- |
|  | uOR [95% CI] |  | uOR [95% CI] |  | uOR [95%CI] |
| **Age group** |  |  |  |  |  |
| 15-19 (ref.) | 1.00 |  | 1.00 |  | 1.00 |
| 20-24 | 2.06***[1.8,2.36] |  | 0.44***[0.37,0.53] |  | 0.56***[0.43,0.71] |
| 25-29 | 3.32***[2.9,3.79] |  | 0.32***[0.27,0.39] |  | 0.51***[0.4,0.65] |
| 30-34 | 5.49***[4.8,6.27] |  | 0.27***[0.22,0.32] |  | 0.51***[0.4,0.65] |
| 35+ | 10.27***[8.99,11.73] |  | 0.12***[0.1,0.15] |  | 0.45***[0.35,0.57] |
| **Parity** |  |  |  |  |  |
| 0 (ref.) | 1.00 |  | 1.00 |  | 1.00 |
| 1 | 4.59***[4.04,5.21] |  | 1.68***[1.36,2.07] |  | 1.23***[0.96,1.58] |
| 2 | 10.67***[9.43,12.08] |  | 0.51***[0.41,0.62] |  | 0.36***[0.28,0.46] |
| 3 | 11.68***[10.31,13.23] |  | 0.34***[0.28,0.42] |  | 0.33***[0.26,0.42] |
| 4+ | 7.86***[6.94,8.9] |  | 0.36***[0.29,0.44] |  | 0.57***[0.45,0.73] |
| **Son child** |  |  |  |  |  |
| Yes (ref.) | 1.00 |  | 1.00 |  | 1.00 |
| No | 0.72***[0.64,0.82] |  | 1.51***[1.46,1.57] |  | 1.32***[1.26,1.38] |
| **Years of schooling** |  |  |  |  |  |
| No education (ref.) | 1.00 |  | 1.00 |  | 1.00 |
| 1-5 years | 1.17***[1.13,1.21] |  | 1.53***[1.47,1.58] |  | 0.94*[0.9,0.99] |
| 6-10 years | 1[0.97,1.03] |  | 2.2***[2.14,2.27] |  | 1.02*[1.01,1.05] |
| 11 & above years | 0.92*[0.89,0.95] |  | 4.22***[4.09,4.36] |  | 1.1[0.31,1.11] |
| **Wealth status** |  |  |  |  |  |
| Poorest (ref.) | 1.00 |  | 1.00 |  | 1.00 |
| poorer | 1.69***[1.64,1.75] |  | 1.18***[1.13,1.23] |  | 0.81***[0.78,0.85] |
| middle | 2.16***[2.08,2.23] |  | 1.02[0.98,1.06] |  | 0.66***[0.63,0.69] |
| richer | 2.1***[2.03,2.17] |  | 1.25***[1.2,1.3] |  | 0.63***[0.6,0.66] |
| richest | 2.08***[2.01,2.16] |  | 2.18***[2.1,2.26] |  | 0.75***[0.71,0.78] |
| **Religion** |  |  |  |  |  |
| Hindu (ref.) | 1.00 |  | 1.00 |  | 1.00 |
| Muslim | 0.57***[0.55,0.59] |  | 2.75***[2.67,2.83] |  | 1.82***[1.75,1.89] |
| Christain | 1.10*[1.02,1.19] |  | 0.59***[0.53,0.65] |  | 0.54***[0.47,0.61] |
| Others | 1.42***[1.33,1.52] |  | 2.05***[1.96,2.16] |  | 0.94[0.87,1.01] |
| **Caste** |  |  |  |  |  |
| General (ref.) | 1.00 |  | 1.00 |  | 1.00 |
| OBC | 0.94***[0.92,0.97] |  | 0.47***[0.45,0.48] |  | 0.73***[0.71,0.76] |
| SC | 1.09***[1.05,1.12] |  | 0.5***[0.49,0.52] |  | 0.77***[0.74,0.8] |
| ST | 0.96*[0.92,1] |  | 0.37***[0.35,0.38] |  | 0.56***[0.53,0.6] |
| Don’t know | 0.82***[0.77,0.87] |  | 1.4***[1.34,1.47] |  | 1.37***[1.28,1.46] |
| **Mass media exposure** | |  |  |  |  |
| No (ref.) | 1.00 |  | 1.00 |  | 1.00 |
| Yes | 1.44***[1.4,1.47] |  | 1.44***[1.41,1.48] |  | 0.82***[0.8,0.85] |
| **Place of residence** |  |  |  |  |  |
| Urban (ref.) | 1.00 |  | 1.00 |  | 1.00 |
| Rural | 0.89***[0.87,0.91] |  | 0.60***[0.59,0.61] |  | 1[0.97,1.03] |
| **Region** |  |  |  |  |  |
| North (ref.) | 1.00 |  | 1.00 |  | 1.00 |
| Central | 0.58***[0.56,0.6] |  | 0.71***[0.69,0.73] |  | 2.11***[2.02,2.2] |
| east | 0.61***[0.59,0.63] |  | 0.88***[0.85,0.9] |  | 1.49***[1.42,1.56] |
| North-east | 0.50***[0.47,0.54] |  | 2.47***[2.34,2.62] |  | 3.58***[3.34,3.83] |
| West | 0.95*[0.91,0.99] |  | 0.43***[0.41,0.44] |  | 0.34***[0.32,0.37] |
| South | 1.67***[1.6,1.74] |  | 0.06***[0.06,0.07] |  | 0.07***[0.06,0.08] |
